# Dynamics of digital ulcers in systemic sclerosis

**DOI:** 10.3892/etm.2020.9127

**Published:** 2020-08-19

**Authors:** Carmen Bobeică, Alin Laurenţiu Tatu, Mihaela Crăescu, Laura Gheuca-Solovăstru

Exp Ther Med 20:61–67, 2020; DOI: 10.3892/etm.2020.8572

After the publication of the above paper, the authors have realized that the last author’s name was spelt incorrectly as “Laura Solovăstru”. The correct spelling (“Laura Gheuca-Solovăstru”) is presented above.

The authors regret that this error was not noticed prior to the publication of their paper, and apologize for any inconvenience caused to the readership of the Journal.

